# Supplementary material for: Modelling Skylarks (Alauda arvensis) to Predict Impacts of Changes in Land Management and Policy: Development and Testing of an Agent-Based Model
Source: PLoS One. 2013 Jun 6;8(6):e65803. doi: 10.1371/journal.pone.0065803 (PMC3675089; doi:10.1371/journal.pone.0065803)
Supplement: Supporting Information S4 — The skylark ODdox as a zipped archive. (ZIP) [file pone.0065803.s004.zip › Skylark_ODdox/struct_pesticide_event.html]

ALMaSS Skylark ODdox: PesticideEvent Struct Reference


ALMaSS Skylark ODdox
 2.0


- Main Page
- Related Pages
- Classes
- Files

- Class List
- Class Index
- Class Hierarchy
- Class Members

Public Member Functions |
Public Attributes

PesticideEvent Struct Reference

`#include <pesticide.h>`

List of all members.

|  |  |
| --- | --- |
| Public Member Functions | |
|  | PesticideEvent (LE \*a\_sprayed\_elem, double a\_amount) |

|  |  |
| --- | --- |
| Public Attributes | |
| double | m\_amount |
| LE \* | m\_sprayed\_elem |

---

## Constructor & Destructor Documentation

|  |  |  |  |
| --- | --- | --- | --- |
| PesticideEvent::PesticideEvent | ( | LE \* | *a\_sprayed\_elem*, |
|  |  | double | *a\_amount* |
|  | ) |  |  |

inline

References m\_amount, and m\_sprayed\_elem.

{

m\_sprayed\_elem = a\_sprayed\_elem;

m\_amount = a\_amount;

}

---

## Member Data Documentation

double PesticideEvent::m\_amount

Referenced by PesticideEvent().

LE\* PesticideEvent::m\_sprayed\_elem

Referenced by PesticideEvent().

---

The documentation for this struct was generated from the following file:

- pesticide.h


- PesticideEvent
- Generated on Thu Jan 10 2013 13:15:36 for ALMaSS Skylark ODdox by
   1.8.1.1
